# Combined Hepatotoxicity and Toxicity Mechanism of Intermedine and Lycopsamine

**DOI:** 10.3390/toxins14090633

**Published:** 2022-09-13

**Authors:** Ziqi Wang, Liang Qiao, Qinqin Zheng, Haolei Han, Zuguang Li, Xiangchun Zhang, Hongping Chen

**Affiliations:** 1 Tea Research Institute, Chinese Academy of Agricultural Sciences, Hangzhou 310008, China; 2 College of Chemical Engineering, Zhejiang University of Technology, Hangzhou 310008, China; 3 The First Affiliated Hospital, College of Clinical Medicine of Henan University of Science and Technology, Luoyang 471003, China; 4 Key Laboratory of Tea Quality and Safety & Risk Assessment, Ministry of Agriculture, Hangzhou 310008, China

**Keywords:** pyrrolizidine alkaloids, intermedine, lycopsamine, tea, combined toxicity, endoplasmic reticulum stress

## Abstract

Pyrrolizidine alkaloids (PAs) are common constituents of plants and have serious hepatotoxicity. Intermedine (Im) and lycopsamine (La) are two monoesters of PAs that frequently coexist in the PA-containing plants (e.g., *comfrey* and tea). The present study aimed to explore the combined hepatotoxicity and toxicity mechanism of the Im and La mixture. In vitro, the combined cytotoxicity of the Im and La mixture on human hepatocytes (HepD) was examined by CCK-8, colony formation, wound healing, and Annexin V/PI staining assays. The combination of Im and La inhibited the ability of HepD cells to proliferate, colonize, and migrate and induced hepatocytes apoptosis in a dose-dependent manner. In addition to significantly causing a burst of intracellular reactive oxygen species (ROS), mitochondrial apoptosis, and endoplasmic reticulum (ER) stress, the Im and La mixture can also cause an increase in intracellular Ca^2+^, triggering the PERK/eIF2α/ATF4/CHOP apoptosis pathway. This study provided the first direct evidence that the combined PAs induced hepatotoxicity through ER-mediated apoptosis. These results supplemented the basic toxicity data for the combined PAs and provided a new perspective for the risk assessment of combined PA toxicity.

## 1. Introduction

Pyrrolizidine alkaloids (PAs) are secondary metabolites in plants and exist in about 3% of the flowering plants worldwide [1,2]. To date, over 660 structurally different PAs and their N-oxide derivatives (PANOs) have been identified in over 6000 plant species of three families: *Boraginaceae*, *Asteraceae*, and *Fabaceae* [3]. Among those, about half of the PAs exhibit toxicity and carcinogenicity [4]. PAs poison grazing animals and domestic livestock through PA-containing plants or feed. PAs poison humans through PA-contaminated food (e.g., grains, honey, and milk); herbal medicine; and plant-derived supplements [5,6]. It was reported that PAs-contaminated food caused chronic liver diseases and hepatic sinusoidal obstruction syndrome (HSOS) in humans [5,7,8,9]. PA-induced HSOS clinically manifests as painful hepatomegaly, ascites, and abdominal distension [10,11]. In 2020, the European Union set the maximum levels of PAs in certain foodstuffs. The limit of PAs in herbal infusions was 200 μg/kg and, in flavored tea, was 150 μg/kg. The limit of PAs in liquid herbal infusions for infants and young children was low to 1.0 μg/kg [12]. The severe toxicity of PAs has attained urgent attention all around the world.

**Figure 1:**
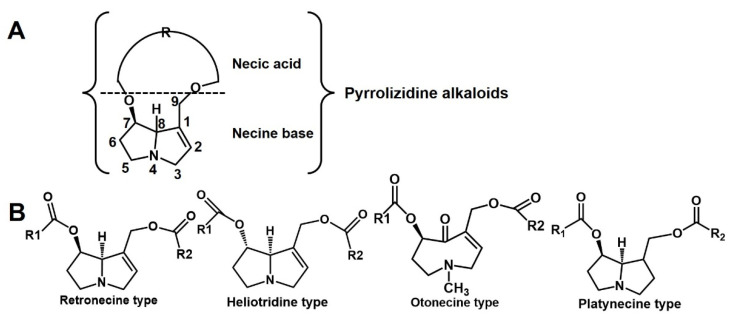
(**A**) The basic structure of PAs. (**B**) PAs are classified as retronecine type, heliotridine type, otonecine type and platynecine type.

PA toxicity is closely related to the structure of PAs. PAs are formed by a necine base (amino alcohol) and one or more necic acid (aliphatic carboxylic acids) (Figure 1A). The double bond in the C1,2 position of necine base is the leading cause of PA toxicity [13]. As shown in Figure 1B, the 1,2-unsaturated PAs are classified into a retronecine type, heliotridine type, and otonecine type. The saturated PAs only consist of the platynecine type. Among the structures of the unsaturated PAs, the retronecine-type and heliotridine-type are diastereomers, and the main difference between them is the structure of the C-7 position. According to the structure of necic acid, the retronecine-type PAs are further divided into a 12-membered cyclic diester, 11-membered cyclic diester, open-ring diester, and monoester [14]. The 1,2-unsaturated PAs require further metabolic activation to exert toxicity. Mediated by a dehydrogenation step, PA is transformed into a highly reactive intermediate dehydropyrrole ester with catalyzing by hepatic cytochrome P450s [15]. Dehydropyrrole esters react with DNA in the nucleus and generate DNA crosslinks or DNA–protein crosslinks, then causing serious liver damage [16]. In addition, active dehydropyrrole esters are also combined with proteins and generate pyrrole–protein adducts in the blood. Pyrrole–protein adducts are the biomarker for PA-induced liver injury [17], and the levels of the pyrrole–protein adducts indicate the potential toxicity of PAs. It was reported that the toxicity of monoesters was lower than diesters in retronecine-type PAs [14]. PAs cause cumulative damage to hepatocytes [18], which will cause liver damage when humans are exposed to monoester PAs for a long time.

In practice, various PAs are usually detected in one kind of sample. About 91% of tea samples have been found to contain one or more PA in the European union market [19]. Our previous research found that intermedine N-oxide, jacobine, jacobine N-oxide, senecionine, senecionine N-oxide, seneciphylline, and senkirkine were detected in tea samples, and the maximum content of them was 151.33 μg/mL [20]. In addition, the copresence of echimidine, echiumine, acetylechimidine, lycopsamine, and intermedine was detected in the honey samples [21]. A large number of studies have reported the toxicity and toxicity mechanism of single PA [22,23,24]. However, no studies have reported the hepatotoxicity of complex PAs. Hence, extensive and in-depth research on the combined toxicity of PAs is urgently desired.

Intermedine (Im) and lycopsamine (La) are monoesters in retronecine-type PAs, and both of them have a high detection rate and high exposure in PA-containing plants. Im and La are epimeric monoesters and are usually detected in one plant [25]. In *Artemisia capillaris Thunb* plants, Im and La are the main PA, and their proportions in the total PA contents were 79.1% and 82.6%, respectively [26]. Meanwhile, Im and La are the predominant PA in *comfrey* plants [27], and intermedine N-oxide and lycopsamine N-oxide are the predominant alkaloids in *Amsinckia* genus plants [25]. In addition, the previous study reported that 100 μg/mL Im had obvious toxicity on human hepatocytes (HepD) and induced HepD cell apoptosis by triggering intracellular reactive oxygen species (ROS) burst and mitochondrial apoptosis [28]. La also had cytotoxicity toward rat primary hepatocytes [18]. Thus, the combined hepatotoxicity and the underlying molecular mechanisms of the Im and La mixture warrant an in-depth evaluation.

In addition to mitochondria-mediated hepatocyte apoptosis, endoplasmic reticulum (ER) stress is also closely related to phytotoxin-induced apoptosis [29,30]. ER is a crucial organelle in cells and is formed by a continuous membranous network of sacs and tubes [31]. Meanwhile, ER is also the main place for the synthesis, folding, and secretion of proteins, including secreted proteins, membrane-bound proteins, and some organelle-targeted proteins. Intracellular ROS burst and calcium (Ca^2+^) overload could cause ER dysfunction and further result in ER stress [31,32]. ER stress leads to the continuous accumulation and aggregation of the unfolded proteins in cells and further induces cytotoxicity [30]. However, whether ER stress is associated with the combined hepatotoxicity of PAs has not been reported.

In this study, we demonstrated the combined effects of Im and La on human hepatocyte (HepD) proliferation, colonization, and migration ability, and the mixture caused significantly cytotoxicity. Meanwhile, the combined PAs induced hepatocytes apoptosis by initiating an intracellular ROS burst, Ca^2+^ overload, mitochondrial membrane potential drops, and mitochondrial structure disruption. Moreover, the mixture of Im and La increased the protein expressions of Bax, caspase-3, caspase-9, and cl-PARP to activate mitochondrial apoptosis. On the other hand, we found that combined Im and La treatments accelerate hepatocytes apoptosis by increasing the related protein expressions of the ER-stress pathway (ATF4 and CHOP). This study provided the first evidence for the toxicity mechanism of the combined PAs being related to ER-mediated apoptosis. This research provided basic toxicity data for the combined PAs and contributed to further knowledge of the true toxicity of plants containing PAs.

## 2. Results

### 2.1. Cytotoxicity of Im and La Mixture on HepD Cells

**Figure 2:**
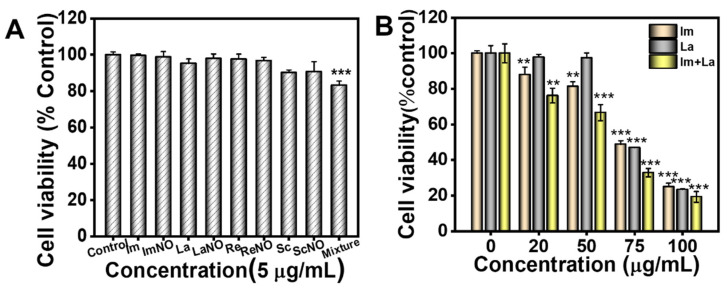
(**A**) The viability of HepD cells after treatment with 5 μg/mL of Im, ImNO, La, LaNO, Re, ReNO, Sc, and ScNO alone or mixed, respectively. (**B**) The viability of HepD cells after treatment with different concentrations (0, 20, 50, 75, and 100 μg/mL) of Im, La, and Im plus the La mixture, respectively. Data are expressed as the mean ± S.D. of three independently repeated experiments. ** *p* < 0.01 and *** *p* < 0.001.

The effect of mixed PAs on the viability of HepD cells was examined by the CCK-8 assay. We assessed the individual or combined toxic effects of eight PAs at a concentration of 5 μg/mL. It was found that a single PA with 5 μg/mL had no obvious toxicity on HepD cells, as indicated in Figure 2A. However, a mixture with eight PAs had a significant cytotoxicity on HepD cells. After treatment with the mixture, the cell viability of HepD cells was decreased to 83.2% compared to the control group. Subsequently, to further assess the cytotoxicity of the combined PAs, we examined the cell viability after treatment with individual PA (Im or La) and combined PA (Im and La mixture), respectively. As depicted in Figure 2B, compared with the control group, the cell viability was 48.8% with the Im treatment at 75 μg/mL and 24.9% at 100 μg/mL. Meanwhile, the cell viability was 47.0% and 23.5% with the treatment of La at 75 and 100 μg/mL, respectively. These data showed that Im or La with a high dose had significant effects on the HepD cell viability. In addition, the cell viability was 32.9% and 19.3% for the treatment of the mixture of Im and La at 75 and 100 μg/mL, respectively. Compared with Im or La, the Im and La mixture had more significant cytotoxicity on the HepD cells. These results indicated that the combined PAs had a more significant inhibition on HepD cell viability than the single PA.

**Figure 3:**
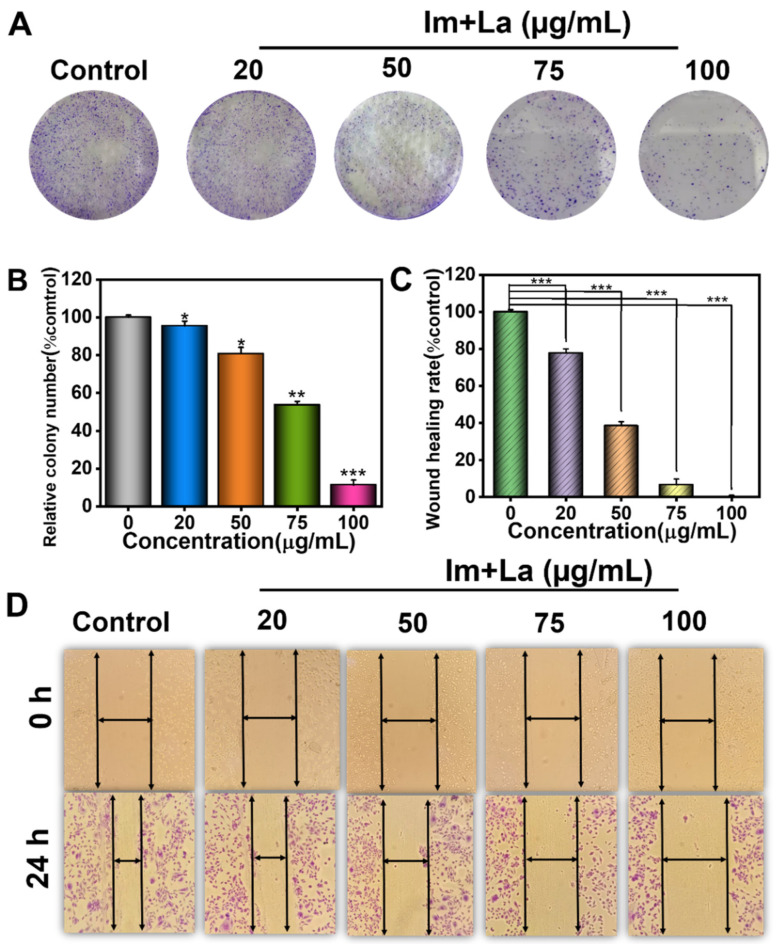
The effects of the Im and La mixture on the colony formation and migration of HepD cells. (**A**,**B**) Representative images and quantification of the colony formation assay of HepD cells. (**C**) The cell migration rates in the cells of five groups were determined by the wound-healing assay. (**D**) The cell migration representative micrographs were taken at 0 h and 24 h. Data are expressed as the mean ± S.D. of three independently repeated experiments. * *p* < 0.05; ** *p* < 0.01; and *** *p* < 0.001.

Since the CCK-8 assay proved that the Im and La mixture exhibited high inhibitory effects on HepD cell proliferation, the cell colony formation assay was further used to evaluate the ability of a single cell to grow into a colony in vitro. HepD cells were treated with different concentrations of the Im and La mixture (0, 20, 50, 75, and 100 μg/mL). As shown in Figure 3A, the Im and La mixture inhibited the long-term proliferation and colonization of HepD cells. Compared with the control group, the ratio of the HepD cell colony formation was 53.6% and 11.3% with the treatment of the Im and La mixture at 75 and 100 μg/mL, respectively (Figure 3B). These data showed that the number of colonies decreased with the increasing concentration of the Im and La mixture. The wound-healing assay further assessed the inhibitory effects of the Im and La mixture on HepD cell migration by detecting the ability of single-cell layers to migrate in vitro [33,34]. HepD cells were treated with the Im and La mixture at a series of concentrations of 0, 20, 50, 75, and 100 μg/mL. Compared with the control group, the Im and La mixture had a significant inhibition of HepD cell migration. The inhibitory effect of the Im and La mixture was increased with the increasing treatment concentrations (Figure 3D). The migration distance of the control was set as 100%, and the wound-healing rates of the HepD cells were 6.7% and 0% with the treatment of the Im and La mixture at 75 and 100 μg/mL, respectively (Figure 3C). The migration ability of HepD cells was completely lost after the treatment with the 100 μg/mL Im and La mixture.

### 2.2. Im and La Mixture Induced Cell Apoptosis

**Figure 4:**
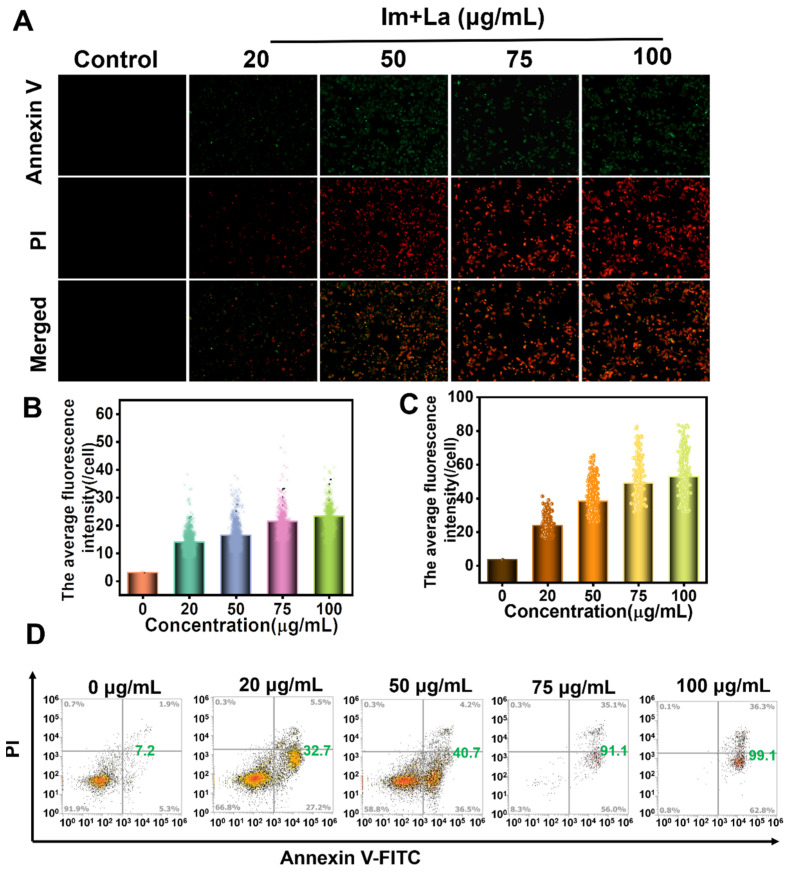
(**A**) Representative fluorescence images of the apoptosis HepD cells were treated with different concentrations of the Im and La mixture for 24 h. (**B**) Green fluorescence intensity statistical data. (**C**) Red fluorescence intensity statistical data. (**D**) The apoptosis rate of HepD cells that were treated with different concentrations of the Im and La mixture for 24 h.

The results of the CCK-8, colony formation, and wound-healing assays have demonstrated that the Im and La mixture had an inhibitory ability on cell proliferation, colony formation, and migration. Here, we further applied the Annexin V/PI double-staining assay and flow cytometry to qualitatively and quantitatively assess the Im and La mixture-induced apoptosis. The Annexin V-FITC probe marked early apoptotic cells with green fluorescence. PI probes penetrated incomplete cell membranes and marked late apoptotic cells, dead cells, or necrotic cells with red fluorescence. HepD cells were treated with the 0, 20, 50, 75, and 100 μg/mL Im and La mixture for 24 h. Cells showed no fluorescence in the PBS treatment group and began to appear weak as green and red fluorescence at the concentration of 20 μg/mL and indicating that HepD cells began apoptosis (Figure 4A–C). The appearance of red fluorescence indicated that cell membranes began to be destroyed. The green and red fluorescence increased obviously in HepD cells at the concentration of 50 μg/mL. Meanwhile, the number of cells with bright green and red fluorescence increased significantly at the concentrations of 75 and 100 μg/mL, indicating that the integrity of the cell membranes was further destroyed. These results indicated that the green and red fluorescence intensity increased with the increasing concentration of the Im and La mixture. The flow cytometry analysis was applied to quantitatively detect the number of 181 apoptotic cells (Figure 4D). The results revealed that the ratio of apoptotic cells elevated as the Im and La mixture concentration increased. The apoptotic rates of the HepD cells treated with 0, 20, 50, 75, and 100 μg/mL Im and La mixture were 7.2%, 32.7%, 40.7%, 91.1%, and 99.1%, respectively. These results disclosed that the Im and La mixture strongly induced HepD cell apoptosis. The rate of apoptotic cells was increased with the treated concentration and was in a concentration-dependent manner.

### 2.3. Im and La Mixture Triggered ROS Burst in HepD Cells

**Figure 5:**
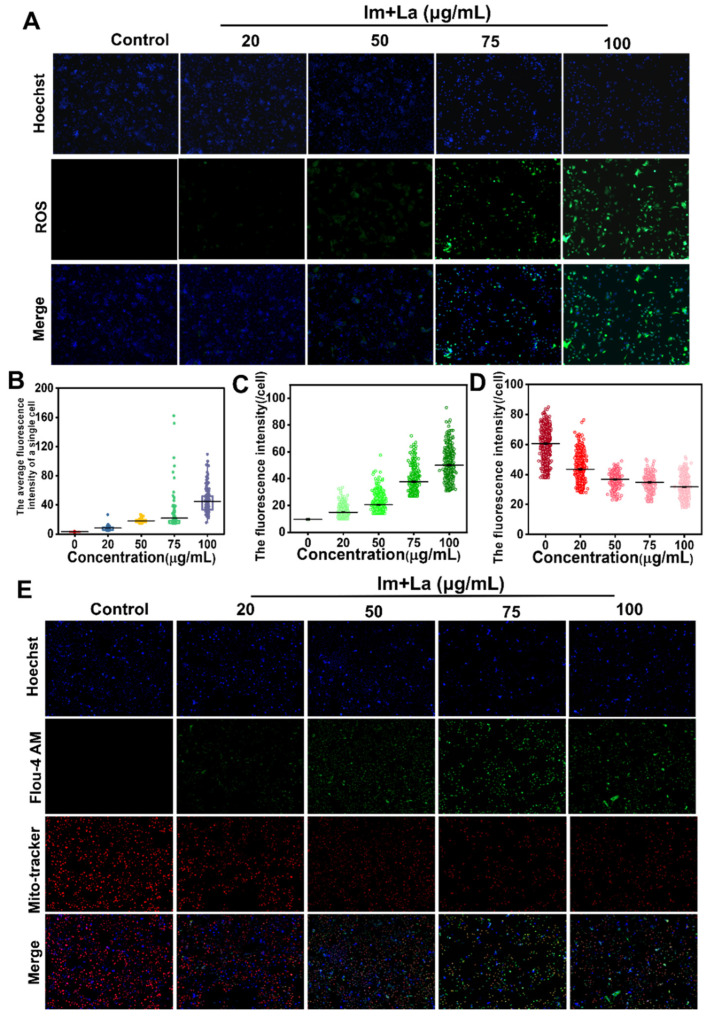
(**A**) Representative pictures of the ROS levels in HepD cells treated by 0, 20, 50, 75, and 100 μg/mL Im and La mixture. (**B**) The quantitative statistics of the ROS green fluorescence intensity. (**C**) The quantitative statistics of the Fluo 4-AM green fluorescence intensity. (**D**) The quantitative statistics of the Mito-Tracker Red fluorescence intensity. (**E**) Representative images of the Ca^2+^ levels and cell apoptosis in HepD cells treated by 0, 20, 50, 75, and 100 μg/mL Im and La mixture for 24 h.

The PA-induced hepatoxicity by causing intracellular oxidative stress damage has been widely reported [24], and oxidative stress causes the overproduction of ROS. To explore whether ROS was involved in the hepatotoxicity induced by the Im and La mixture, we used the DCFH-DA probe to detect the levels of intracellular ROS after Im and La mixture treatment. HepD cells were incubated with 0, 20, 50, 75, and 100 μg/mL the Im and La mixture, respectively. As shown in Figure 5A,B, HepD cells showed weak green fluorescence for the treatment at 50 μg/mL in comparison to the control. The green fluorescence intensity reflected the number of intracellular ROS. The green fluorescence intensity was significantly increased at the concentrations of 75 μg/mL and 100 μg/mL Im and La mixture. These results showed that the green fluorescence intensity and numbers significantly increased with the increasing concentration of the Im and La mixture and indicated that the Im and La mixture significantly enhanced the levels of intracellular ROS. Combined with the results of the flow cytometry analysis, HepD cell apoptosis caused by the Im and La mixture was tightly related to intracellular ROS overproduction.

### 2.4. Im and La Mixture Elevated Intracellular Calcium Levels

Ca^2+^ is the vital second messenger in intracellular signal delivery. The intracellular Ca^2+^ levels are related to the physiological activities of cells and cell death [35]. To explore the Ca^2+^ levels whether related to Im and La mixture-induced hepatocytes apoptosis, we used a Fluo 4-AM probe to detect the intracellular Ca^2+^ levels. HepD cells were treated with different concentrations (0, 20, 50, 75, and 100 μg/mL) of the Im and La mixture for 24 h. As shown in Figure 5C, the green fluorescence intensity was increased significantly with the increasing concentrations of the Im and La mixture. The weak green fluorescence appeared in the 50-μg/mL dose group, and the intensity of the green fluorescence significantly increased for the Im and La mixture treatment at 75 and 100 μg/mL, respectively. These results revealed that the Im and La mixture increased the intracellular Ca^2+^ levels in a dose-dependent manner. According to the results of the Annexin V/PI assay, the Im and La mixture caused HepD cell apoptosis and was related to the intracellular Ca^2+^ overload.

### 2.5. Im and La Mixture Destroyed Mitochondrial Structure

**Figure 6:**
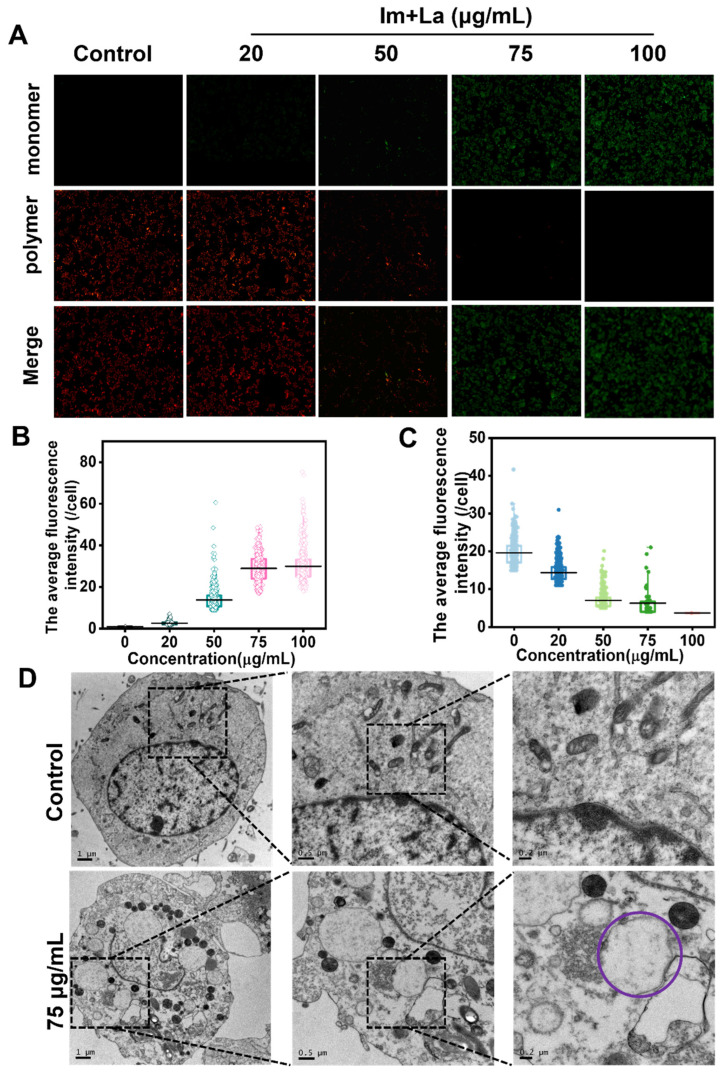
(**A**) The JC-1 green/red fluorescence images of HepD cells were treated with 0, 20, 50, 75, and 100 μg/mL Im and La mixture. (**B**) The statistical graph of JC-1 green fluorescence intensity of (**A**). (**C**) The statistical graph of JC-1 red fluorescence intensity of (**A**). (**D**) TEM images about mitochondrial morphological changes in HepD cells after 0 and 75 μg/mL Im and La mixture treatment for 24 h. Scale bar = 1 μM, 0.5 μM, 0.2 μM.

Mitochondria are important organelles and play a crucial role in regulating cell physiological activities. Mitochondria are closely related to cell apoptosis and the regulation of intracellular ROS and the Ca^2+^ levels [36]. To detect whether mitochondria were related to Im and La mixture-induced HepD cells apoptosis, the Mito-Tracker Red CMXRos probe was used to detect the physiological state of the mitochondria. Mito-Tracker Red CMXRos probes depend on the mitochondrial membrane potential to specifically label active mitochondria. The HepD cells were treated with the Im and La mixture (0, 20, 50, 75, and 100 μg/mL) for 24 h. As shown in Figure 5D,E, the red fluorescence intensity was reduced gradually with the treatment of the Im and La mixture. The red fluorescence was extremely reduced with the treatment of the Im and La mixture at 50 μg/mL, with almost no red fluorescence for the treated concentrations at 75 and 100 μg/mL, respectively. It was indicated that the Im and La mixture caused mitochondrial structural disruption. The decrease of the mitochondrial inner membrane potential marks the occurrence of cell apoptosis [37]. The results of this experiment indicated that the Im and La mixture induced cell apoptosis. We further used JC-1 probes to detect the decrease of the mitochondrial inner membrane potential. JC-1 probes could mark the mitochondrial inner membrane potential. JC-1 probes aggregate and generate a red polymer at a high mitochondrial membrane potential. JC-1 probes are green monomers when the mitochondrial membrane potential is low [38]. HepD cells were treated with different concentrations (0, 20, 50, 75, and 100 μg/mL) of the Im and La mixture for 24 h; then, we used the fluorescence microscope to observe cells after staining with JC-1 dye. The bright green fluorescence appeared for the treated concentration at 75 and 100 μg/mL, respectively (Figure 6A,B), while the red fluorescence was significantly reduced with the increasing concentration (Figure 6A,C). These results on the statistical graph of the red and green fluorescence intensity indicated that the Im and La mixture reduced the mitochondrial membrane potential. On the other hand, the changes in the mitochondrial morphology with the Im and La mixture treatment were observed by TEM. In Figure 6D, compared with the control group, most of the mitochondria shrunk and gathered around the nucleus in cells after the Im and La mixture treatment at 75 μg/mL. The nucleus appeared to be ruptured, and a few mitochondria showed cysts with the treatment of the Im and La mixture (purple circle in Figure 6D). These TEM images directly showed that the combined Im and La treatment caused the mitochondrial structure to be damaged, which further led to the impairment of mitochondrial function and caused cell necrosis.

### 2.6. Im and La Mixture Caused ER-Mitochondria Colocalization and Triggered Hepatocyte Apoptosis

**Figure 7:**
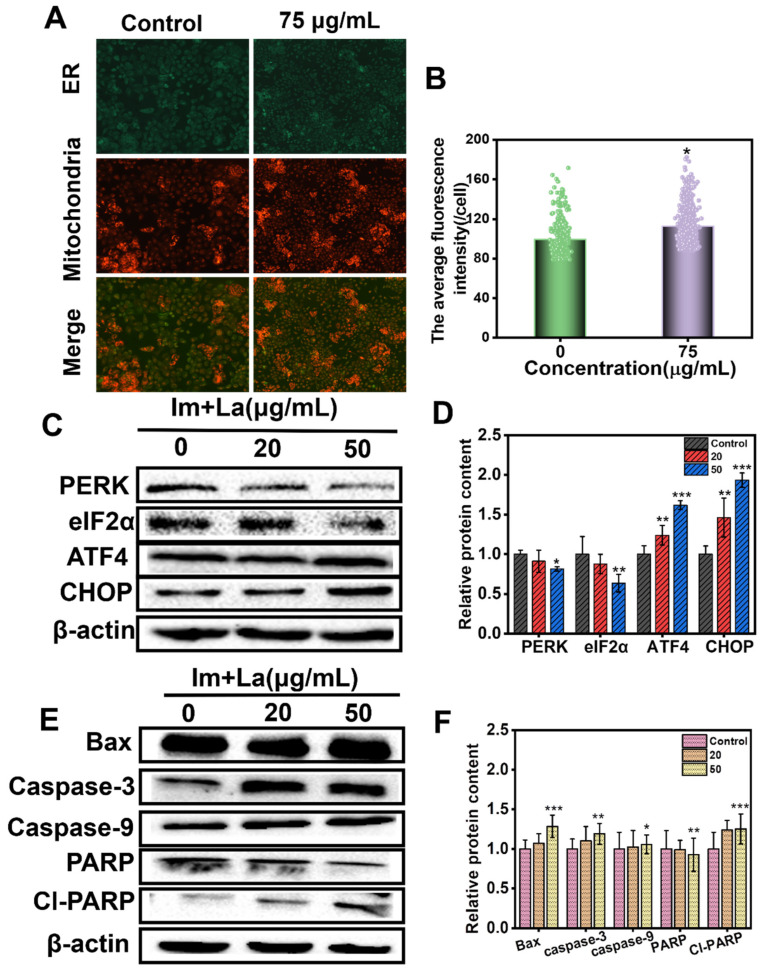
(**A**) The ER and mitochondria membrane crosslinking images of HepD cells. Cells were treated with 0 and 75 μg/mL Im and La mixture for 24 h and marked with Mito-Tracker Red probes and ER-Tracker Green probes. (**B**) The statistical chart of the ER and mitochondria membrane crosslinking areas. (**C**) The protein expression levels of PERK, eIF2α, ATF4, and CHOP in HepD cells. Cells were treated with 0, 20, and 50 μg/mL Im and La mixture for 24 h. (**D**) The quantitative Western blot results of PERK, ATF4, eIF2α, and CHOP. (**E**) The protein expression levels of Bax, caspase-3, caspase-9, PARP, and cl-PARP in HepD cells were treated with 0, 20, and 50 μg/mL Im and La mixture for 24 h. (**F**) The quantitative Western blot results of Bax, caspase-3, caspase-9, PARP, and cl-PARP. The data were presented as the mean ± S.D., n = 3. *: *p* < 0.05, **: *p* < 0.01, and ***: *p* < 0.001.

The overload of Ca^2+^ could cause functional damage to the ER and mitochondria. Ca^2+^ are secondary signaling molecules, and the ER is an important organelle to store Ca^2+^ in the cytoplasm. The ER is a crucial organelle for regulating cellular physiological activities [35]. The transportation of Ca^2+^ between the ER and mitochondria requires the membrane fusion of two organelles [39]. Mito-Tracker Red fluorescent probes could mark the mitochondria, and ER-Tracker Green probes mark the ER. As shown in Figure 7A,B, compared to the control, the area of mitochondria and ER colocalization became larger after 75 μg/mL Im and La mixture treatment. These results showed that the Im and La mixture induced the crosslink of the ER and mitochondria, and changes in the membrane structure in the ER and mitochondria happened.

The membrane structure changing is the early sign of mitochondria apoptosis. The mitochondrial-mediated pathway is an endogenous pathway that causes cell apoptosis [15]. Based on the results of the Im and La mixture destroying the mitochondrial structure, we further examined the expression of mitochondrial-mediated apoptosis pathway proteins, including Bax, caspase-3, caspase-9, and RARP. When the apoptosis signal entered cells, the proapoptotic protein (Bax) was activated. The permeability of the mitochondrial membrane was increased, and cytochrome c (Cyt c) was released from the mitochondria into the cytoplasm. In the cytoplasm, Cyt c activated the expression of the caspase-9 protein. Phosphorylated caspase-9 bonded with the apoptotic protease activating factor (Apaf1), generating apoptosome. Next, the apoptosome activated caspase-3 to start the apoptosis process. Poly ADP-ribose polymerase (PARP) was their substrate, and the presence of cleaved PARP demonstrated that caspase-3 was activated. The apoptosis process was started [15,40]. As depicted in Figure 7E,F, after treatment of the Im and La mixture for 24 h, the expression of Bax, caspase-3, caspase-9, and cl-PARP was upregulated in HepD cells in a concentration-dependent manner, while the protein expression of PARP was downregulated with the increasing concentration of the Im and La mixture. Caspase-9 protein is the initiator of the apoptosis program, and the executor of cell death is caspase-3. The increased expression of caspase-3 and caspase-9 revealed that the caspase-dependent apoptotic pathway was involved in the combined PA-induced apoptosis of HepD cells.

On the other hand, we first found that ER was related to Im and La mixture-induced apoptosis. The expression of ER stress-related proteins was examined with the Western blotting assay, including PERK, eIF2α, ATF4, and CHOP. PERK is pancreatic ER kinase or PKR-like ER kinase, the active PERK phosphorylates eukaryotic initiation factor 2 (eIF2α). Then, the phosphorylated eIF2α activates the translation of transcription factor 4 (ATF4). Next, ATF4 induces the expression of C/EBP homologous protein (CHOP). CHOP is an important proapoptotic protein, and the expression of CHOP initiates apoptosis [30,32]. HepD cells were treated with 0, 20, and 50 μg/mL Im and La mixture for 24 h. The expression of PERK and eIF2α proteins was downregulated at 20 μg/mL and further reduced at 50 μg/mL. However, the levels of ATF4 and CHOP expression were markedly elevated in HepD cells when treated with the 20 μg/mL and 50 μg/mL Im and La mixtures (Figure 7C,D). These results indicated that the Im and La mixture induced ER stress through the PERK/eIF2α/ATF4 /CHOP pathway.

## 3. Discussion

Various PAs were generally detected in PAs-containing foods and plants. Im and its epimer (La) usually coexisted in one plant, and previous research has proved the individual cytotoxicity of Im or La [18,28]. In our study, we found eight retronecine-type PA mixtures at a very low concentrations had higher cytotoxicity than individual PAs at a low concentration (Figure 2). The results showed that the combined toxicity of the PAs was higher than the toxicity of individual PAs. Given that monoester PAs have a high detection rate and exposure level, the mixture of monoester PAs drastically increased the risk of PAs. Therefore, we explored the combined toxicity and toxicity mechanisms of the Im and La mixture in the study.

The results of the CCK-8 assay showed that the Im and La mixture had significant inhibitory effects on the viability of human hepatocytes (HepD). Compared to individual Im or La, the Im and La mixture had higher cytotoxicity on HepD cells (Figure 2). Meanwhile, the cell colony formation assay and the cell wound-healing assay proved that the Im and La mixture severely affected the colonization and migration ability of HepD cells in vitro (Figure 3). The results of these experiments directly suggested that the Im and La mixture markedly inhibited the proliferation, colonization, and migration ability of HepD cells.

In addition, the Im and La mixture induced a significant apoptosis of HepD cells. As shown by the Annexin V/PI double-staining experiment (Figure 4A–C), cell apoptosis was not observed in the control group. In contrast, a large of HepD cells were killed by the treatment of the Im and La mixture and observed with the intensive red and green fluorescence signals. Meanwhile, the results of the flow cytometry proved that the number of apoptotic cells increased after incubation with the Im and La mixture in a concentration-dependent manner. These data all confirmed the Im and La mixture markedly caused cell apoptosis. We speculated that the reason for the results was the Im and La mixture had synergistic toxicity effects on HepD cells. The concept of synergistic toxicity effects was widely used for the environmental health risk assessment of combined exposure to pesticides [41]. More research is needed to further explore the risk of combined exposure to PAs.

Lots of previous research has proven that the molecular mechanisms of PA-induced hepatotoxicity trigger intracellular ROS burst and mitochondria-mediated apoptosis [15,24,40]. ROS are the products of normal metabolism in intracellular mitochondria, the CYP450 system, peroxisomes, and inflammatory cells [42,43]. The generated ROS are eliminated by intracellular antioxidant enzymes [44]. When exogenous substances disrupt the balance of intracellular redox reactions and cause the excessive production of ROS, the over-productive ROS bond to macromolecules (DNA, RNA, proteins, or lipids), then cause cellular death [13]. We found that the concentrations of 75 μg/mL and 100 μg/mL Im and La mixture caused a significant increase in the ROS levels (Figure 5A). The green fluorescence intensity statistic graph intuitively reflected the increase in fluorescence intensity (Figure 5B). Therefore, these results showed that ROS overproduction was related to the apoptosis induced by the Im and La mixture.

On the other hand, the present study demonstrated that the Im and La mixture induced HepD cell apoptosis through mitochondrial-mediated apoptosis. When apoptosis signals were delivered to the mitochondria in the early stages of apoptosis, the membrane permeability of the mitochondria was disrupted [45,46]. Then, electrochemical potential energy was stored in the inner mitochondrial membrane and was released into the cytoplasm [37,38]. The increased green fluorescence intensity and the decreased red fluorescence intensity revealed that the Im and La mixture caused a drop in the mitochondrial membrane potential (Figure 6A–C). Following, we used TEM to observe the morphology of the mitochondria after the Im and La mixture treatment. From the microscopic images, we found that the mitochondria were spherical, and the intact mitochondrial structure was disrupted in the 75 μg/mL treatment group. This image intuitively indicated the occurrence of mitochondrial structural damage. Meanwhile, a Mito-Tracker Red CMXRos probe was used to detect the physiological status of the cell, and the decreased red fluorescence intensity in Figure 5D demonstrates the occurrence of cell apoptosis. Further studies have revealed that the combined PA-induced apoptosis was tightly related to the Bax/caspase-3/caspase-9/cl-PARP pathway in mitochondria. The Western blotting assay proved that the protein expression of Bax, caspase-3, caspase-9, and cl-PARP were all increased. The mitochondria-mediated apoptosis was the underlying molecular mechanism of the combined PA-induced hepatotoxicity.

In addition, the present study, for the first time, directly demonstrated that the Im and La mixture induced hepatoxicity through ER-mediated apoptosis. The Western blotting assay proved ER stress induced by the combined PAs. The expression of the ER-stress-related proteins (PERK, eIF2α, ATF4, and CHOP) was affected. Therein, HepD cells were treated with 0, 20, and 50 μg/mL Im and La mixtures. After the treatment of the Im and La mixtures, the expressions of PERK and eIF2α were decreased, and the expressions of ATF4 and CHOP were increased obviously (Figure 7C,D). The phosphorylated eIF2α increased the translation of the activating transcription factor 4 (ATF4), which is a member of the CCAAT/enhancer-binding protein (C/EBP) family of transcription factors [35,39]. Finally, the active ATF4 protein increased the expression of the downstream protein-CHOP and induced ER stress-mediated apoptosis [30]. These results indicated that ER stress-mediated apoptosis was involved with the Im and La mixture-induced hepatotoxicity.

Meanwhile, we also found intracellular Ca^2+^ was related to the apoptosis induced by the Im and La mixture. Intracellular Ca^2+^ is the ubiquitous secondary messenger, and Ca^2+^ is involved in numerous physiological activities [35]. The results of the Ca^2+^ fluorescence detection assay proved that the intracellular Ca^2+^ levels were significantly increased by the Im and La mixture in a concentration-dependent manner (Figure 5E). Ca^2+^ homeostasis is closely related to the normal function of the mitochondria and ER [47]. Therefore, the damage to the mitochondria and ER caused Ca^2+^ in the organelles to flow into the cytoplasm, then caused Ca^2+^ overload in the cytoplasm. Ca^2+^ overload may act as a potentiation loop for apoptosis, which was related to Im and La mixture-induced hepatocyte apoptosis [36]. Moreover, compared with the control, the crosslinked areas of the ER and mitochondria were increased after the treatment with the 75 μg/mL Im and La mixtures. We speculated that the release of Ca^2+^ from ER stores caused Ca^2+^ overload in the mitochondria through the ER–mitochondria crosstalk and further caused mitochondrial dysfunction. The underlying mechanism of Ca^2+^ overload required further experiments to explore.

## 4. Conclusions

In summary, we confirmed the toxicity of the Im and La mixture on HepD cells, and the hepatotoxicity was directly related to the intracellular ROS burst and the Bax/caspase-3/caspase-9/cl-PARP mitochondrial apoptosis pathway. Additionally, the previous study found monocrotaline induced hepatotoxicity with ER stress [48]. Compared to this, we observed that the Im and La mixture caused Ca^2+^ overload in the cytoplasm and induced the ER-mediated PERK/eIF2α/ATF4/CHOP apoptosis pathway, which had not been reported in previous PA toxicity studies. Based on these findings, we believed that ER-mediated apoptosis was the new crucial step in PA mixture-induced hepatotoxicity. Our study provided the basic theory for evaluating the toxicity of combined PAs.

## 5. Materials and Methods

### 5.1. Chemicals and Reagents

**Figure 8:**
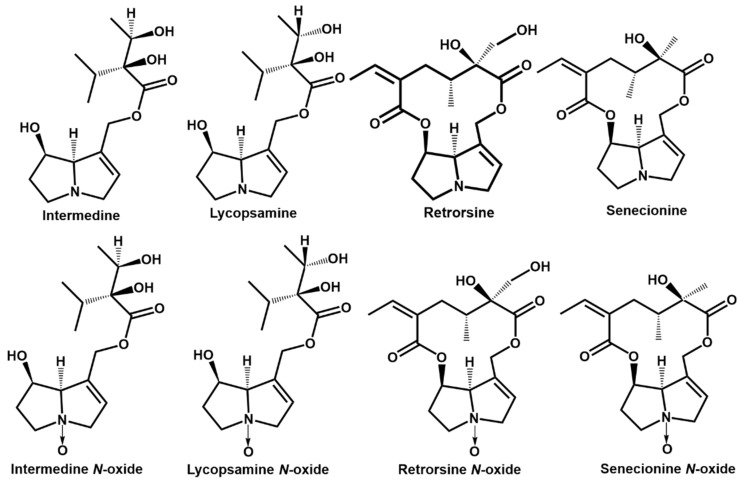
Structures of intermedine (Im), lycopsamine (La), retrorsine, senecionine, intermedine N-oxide, lycopsamine N-oxide, retrorsine N-oxide, and senecionine N-oxide.

PAs were purchased from Standards Biotechnology Co., Ltd. (Shanghai, China), including Im, La, retrorsine (Re), senecionine (Sc), intermedine N-oxide (ImNO), lycopsamine N-oxide (LaNO), retrorsine N-oxide (ReNO), and senecionine N-oxide (ScNO). Their purity was more than 95%. The structures of these compounds are shown in Figure 8.

### 5.2. Cell Culture

**Figure 9:**
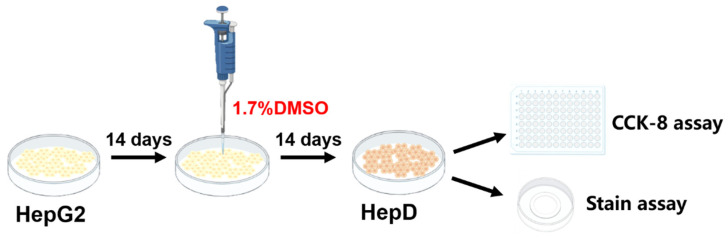
The incubation steps for HepD cells. HepG2 cells were incubated with MEM (10% FBS and 1% streptomycin–penicillin) medium for 14 days and were further differentiated with 1.7% DMSO for another 14 days, and HepG2 cells transformed into human hepatocytes (HepD).

The human hepatocellular carcinoma cell line (HepG2) was obtained from X-Y Biotechnology Co., Ltd. (Shanghai, China). HepG2 cells were proliferated in minimum essential medium (MEM, Hyclone, Thermo Scientific, Waltham, MA, USA) supplemented with 10% (*v*/*v*) fetal bovine serum (FBS, Gibco, Waltham, MA, USA), 100 U/mL penicillin, and 100 μg/mL streptomycin (Gibco, Waltham, MA, USA). As shown in Figure 9, HepG2 cells were incubated in MEM medium supplemented with 10% FBS and 1% antibiotics (100 U/mL penicillin and 100 μg/mL streptomycin) for 14 days. After the proliferation phase, cell differentiation was initiated by adding 1.7% dimethyl sulfoxide (DMSO, Sigma, St. Louis, MO, USA). After the differentiation for 14 days, the cell morphology changed and was polygonal, which resembled normal human hepatocytes (HepD) [49,50]. All experiments were conducted with differentiated cells (HepD).

### 5.3. Cell Viability Assay

The viability of HepD cells after treatment was evaluated by the colorimetric CCK-8 assay. Briefly, after 14 days of differentiation, HepD cells at a density of 2 × 10^4^/well were seeded in the inner 60 wells of the 96-well plate. After incubation of 24 h, in one plate, cells were treated with the individual or mixtures of Im, La, Re, Sc, ImNO, LaNO, ReNO, and ScNO at the concentration of 5 μg/mL for each PA and PBS treatment (Hyclone, Waltham, MA, USA) as the control. In addition, the cells were treated with different concentrations (20, 50, 75, and 100 μg/mL) of single PA (Im or La) and the combined PAs (Im and La mixture; the ratio of Im and La is 1:1), with PBS treatment as the control. After incubation for 24 h, the medium was removed, and 10 μL of CCK-8 reagent (Dojindo, Kumamoto, Japan) was added per well in the plate. The plate was incubated for 30 min at 37 °C, and the absorbance of living cells at 450 nm was recorded using a microplate reader (SpectraMAX M2, Sunnyvale, CA, USA). The absorbance values reflected the cell viability of each group. The cell viability was calculated by a percentage of the control group, and the PBS treatment group was set to 100%. The experiment was repeated three times.

### 5.4. Colony Formation Experiment

HepD cells were seeded in a 6-well plate at a density of 500 cells/well. Cells were cultured in the incubator at 37 °C, with 5% CO_2_ for 7 days, and the medium was changed every 2 days in each well. Next, cells were treated with 0, 20, 50, 75, and 100 μg/mL Im and La mixture, respectively. After incubation overnight, the culture medium was aspirated. Then, 4% paraformaldehyde (Macklin, Shanghai, China) was utilized to fix cells, and cells were stained with 0.1% crystal violet (Beyotime, Shanghai, China). The colony numbers in each well were observed and counted under an inverted optical microscope (Olympus, Tokyo, Japan). The experiment was repeated three times.

### 5.5. Wound Healing Assay

The wound-healing assay was employed to assess cell motility. HepD cells were grown in a 6-well plate at a density of 1 × 10^6^ cells/well and were cultured with a medium for 24 h. When cells reached 90% confluence, the wound was made with the 200-μL pipette tip in the cell monolayer. Then, cells were treated with the Im and La mixture (0, 20, 50, 75, and 100 μg/mL, respectively) and incubated for 24 h. Then, cells were fixed with 4% paraformaldehyde and stained with 0.1% crystal violet for 30 min. The representative images of 0 h and 24 h were obtained under a light microscope (Olympus, Tokyo, Japan), and the wound width was measured using ImageJ software. The experiment was repeated three times.

### 5.6. Annexin V/PI Staining Assay

The Annexin V/propidium iodide (PI) double-staining assay was employed to detect cell apoptosis. HepD cells (2 × 10^5^ cells/well) were seeded in the laser confocal glass-bottom culture dish and incubated for 24 h. Then, cells were treated with the Im and La mixture (0, 20, 50, 75, and 100 μg/mL, respectively) for 24 h. According to the protocol of the Annexin V-FITC/PI cell apoptosis detection kit (Beyotime, Shanghai, China), Annexin V-FITC (10 μL) solution and PI (5 μL) dye were added to the cell culture dish and incubated for 30 min in the dark at 37 °C. Then, the fluorescence images were obtained with a fluorescence microscope (Olympus Corporation, Tokyo, Japan).

### 5.7. Flow Cytometry

Flow cytometry was used to quantitatively detect the apoptosis rate of the cells. Briefly, HepD cells were seeded in a 60-mm culture dish at a density of 1.0 × 10^6^ cells/well and incubated in MEM (10% FBS and 1.7% DMSO) medium at 37 °C in a 5% CO_2_ atmosphere. Then, the cells were treated with 0, 20, 50, 75, and 100 μg/mL Im and La mixture for 24 h. After being washed with PBS three times, the cells were stained by Annexin V-FITC/PI dye. The number of the apoptotic cells was analyzed by flow cytometry (Thermo Attune, Waltham, MA, USA).

### 5.8. Intracellular ROS Levels Detection

The intracellular ROS levels were marked using a DCFH-DA fluorescent probe (Beyotime, Shanghai, China). In brief, HepD cells at a density of 2 × 10^5^ per well were grown in a laser confocal glass-bottom culture dish and incubated for 24 h. HepD cells were treated with the Im and La mixture (0, 20, 50, 75, and 100 μg/mL, respectively) for 24 h. Then, the cells were stained with 5 μL DCFH-DA green fluorescent probes and 0.2 μL Hoechst 33342 nuclear blue fluorescent probes (Beyotime, Shanghai, China) at 37 °C for 30 min in the dark. Finally, the cells were washed with PBS twice, and the intracellular ROS levels were imaged using a fluorescence microscope (Olympus Corporation, Tokyo, Japan). The fluorescence intensity was analyzed with ImageJ software.

### 5.9. Intracellular Calcium Concentration Detection

The cytoplasmic Ca^2+^ concentration was related to ER stress and mitochondria apoptosis, and the increase of Ca^2+^ occurred at the early and late stages of the apoptotic pathway [36] To determine the Ca^2+^ levels in cells, HepD cells were seeded in a laser confocal glass-bottom culture dish at a density of 2 × 10^5^ and incubated for 24 h up to 80% confluence. Then, the cells were treated with the Im and La mixture (0, 20, 50, 75, and 100 μg/mL, respectively) for 24 h. Next, the cells were incubated with 5 μM Fluo-4 AM fluorescent probes (Beyotime, Shanghai, China), 0.2 μL Hoechst 33342 nuclear blue fluorescent probes, and 50 nM Mito-Tracker Red CMXRos probes at 37 °C for 30 min. The intracellular Ca^2+^ levels and mitochondrial apoptosis were imaged using the fluorescence microscope (Olympus Corporation, Tokyo, Japan). The fluorescence intensity was analyzed with ImageJ software.

### 5.10. ER–-Mitochondria Colocalization

The membrane crosslinking between the ER and mitochondria is the structural basis of Ca^2+^ transportation [39]. The fluorescent assay was used to explore the crosslinking between the ER and mitochondria. HepD cells were seeded in the laser confocal glass-bottom culture dish at a density of 2 × 10^5^. HepD cells were treated with the Im and La mixture (0, 75 μg/mL) for 24 h. Then, cells were stained with 50 nM ER-Tracker Green probes (Beyotime, Shanghai, China) and 50 nM Mito-Tracker Red CMXRos probes (Beyotime, Shanghai, China) for 30 min at 37 °C in the dark. Finally, the fluorescent images of the cells were obtained by a fluorescence microscope (Olympus Corporation, Tokyo, Japan). The fluorescence areas were analyzed with ImageJ software.

### 5.11. Mitochondrial Membrane Potential Detection

JC-1 probe was used to detect the changes in the mitochondrial membrane potential [38]. HepD cells were seeded in a glass-bottomed culture dish at a density is 2 × 10^5^/mL and were incubated with 0, 20, 50, 75, and 100 μg/mL Im and La mixture for 24 h. After that, the HepD cells were washed with PBS three times and incubated with JC-1 solution (10 μg/mL; Beyotime, Shanghai, China) for 30 min at 37 °C. Then, the stained cells were imaged by the fluorescence microscope (Olympus Corporation, Tokyo, Japan). The fluorescence intensity was analyzed with ImageJ software.

### 5.12. Cell Morphological Observation

A transmission electron microscope (TEM) was used to observe the cell ultrastructure. We used TEM to observe the structure of the mitochondria in cells. HepD cells were seeded in a 60-mm culture dish at a density of 1 × 10^6^ and treated with 0 and 75 μg/mL Im and La mixtures for 24 h. Then, the cells were digested by 0.25% trypsinization-EDTA and washed with ice-cold PBS twice. Cell pellets were obtained by centrifugation and were fixed with 2.5% glutaraldehyde–PBS buffer for 12 h at 4 °C. Next, cell pellets were washed with PBS three times and were fixed in 1% osmic acid for 2 h. Then, the samples were dehydrated in a graded series of ethanol (50–70–90–95–100%). Cells were embedded in epoxy resins. The samples were sliced with an ultramicrotome and stained with uranyl acetate. Finally, the structures of the mitochondria were observed and imaged with TEM (Talos F200C, FEI, Hillsboro, OR, USA).

### 5.13. Western Blotting Analysis

HepD cells were seeded in a 6-well plate at a density of 2 × 10^5^ cells/well and incubated overnight. Then, cells were treated with 0, 20, and 50 μg/mL Im and La mixtures for 24 h. After treatment, the cells were washed with precooled PBS twice. Protein samples were collected using the RIPA lysis buffer (Beyotime, Shanghai, China). The cell proteins were collected by centrifugation at 12,000× r/min for 15 min at 4 °C. The concentrations of the proteins were measured by the BCA assay kit (Beyotime, Shanghai, China). Then, the samples were mixed with 5× loading buffer and heated at 100 °C for 5 min. Samples with equal protein amounts were run on 10% SDS-PAGE (Beyotime, Shanghai, China) and transferred to a 5% nonfat milk-blocked PVDF membrane for one hour and were incubated with the primary antibodies as follows: Bax, caspase-3, caspase-9, PARP, cl-PARP, eIF2α, CHOP, PERK, and ATF4 (Abcam, Cambridge, UK) overnight at 4 °C. The membranes were incubated at 37 °C for 1.5 h with the horseradish peroxidase-conjugated secondary antibodies anti-rabbit IgG (Abcam, Cambridge, UK) and horseradish peroxidase-conjugated secondary antibodies anti-mouse IgG (Abcam, Cambridge, UK). Finally, the bands were visualized with an ECL Western blot detection reagent (Pierce Biotechnology, Rockford, IL, USA). Representative bands were gained from three independent experiments, and the protein levels were analyzed by ImageJ software.

### 5.14. Statistical Analysis

The experimental results were presented as the mean ± standard deviation (S.D.) of three independent experiments. Statistical analysis was performed using Origin software (version 8.0). One-way ANOVA was applied to evaluate the statistical difference between the treatments. *p*-value < 0.05 (*) was considered a statistically significant difference. *p*-value < 0.01 (**) and *p*-value < 0.001 (***) were considered as highly significant differences.

## Data Availability

The data are contained within the article.

## Abbreviations

PARP	poly ADP-ribose polymerase
cl-PARP	cleaved poly ADP-ribose polymerase
CHOP	C/EBP homologous protein antibody
eIF2α	eukaryotic initiation factor 2α
ATF4	activating transcription factor 4
PERK	Protein kinase R-like endoplasmic reticulum kinase
PBS	Phosphate-buffered saline
